# Multiple System Atrophy (MSA) and smoking: a meta-analysis and mechanistic insights

**DOI:** 10.18632/aging.104021

**Published:** 2020-11-07

**Authors:** Fan-Shuen Tseng, Xiao Deng, Yi-Lin Ong, Hui-Hua Li, Eng-King Tan

**Affiliations:** 1 Yong Loo Lin School of Medicine, National University of Singapore, Singapore 119228, Singapore; 2 Department of Neurology, National Neuroscience Institute, Singapore 169856, Singapore; 3 Department of Clinical Research, Singapore General Hospital, Singapore 169856, Singapore; 4 Duke-NUS Medical School, Singapore 169857, Singapore

**Keywords:** multiple system atrophy, smoking, movement disorders

## Abstract

Background: The association between cigarette smoking and multiple system atrophy (MSA) has been debated. We conducted a systematic review and a meta-analysis to investigate this link.

Results: We identified 161 articles from database searching and bibliographic review. Five case-control studies satisfied the inclusion and exclusion criteria, and 435 and 352 healthy controls and MSA patients were examined. The prevalence of MSA amongst ever smokers was lower compared to never smokers (aOR=0.57; 95% CI, 0.29-1.14), although this result did not reach statistical significance. This was also observed for current and former smokers, with a stronger association for current smokers (aOR=0.63 vs aOR=0.96).

Conclusions: There is a suggestion that smoking protects against MSA. Prospective studies in larger patient cohorts are required to further evaluate the cause-effect relationship and functional studies in cellular and animal models will provide mechanistic insights on their potential etiologic links.

Methods: PubMed and Cochrane Library were searched from inception to July 7, 2019 to identify case-control studies that analyzed smoking as an environmental risk or protective factor for MSA. Two authors independently extracted data and performed risk-of-bias and quality assessment. The random-effects model was assumed to account for between-study variance when pooling the crude and adjusted odds ratios.

## INTRODUCTION

Multiple System Atrophy (MSA) refers to a group of neurodegenerative disorders characterized by autonomic dysfunction, cerebellar abnormalities, parkinsonism and corticospinal degeneration [1]. Its prevalence is estimated to be 2-5 in 100,000, and its rarity limits efforts to better understand the disease, especially its pathogenesis and risk factors [2–5]. MSA belongs to a larger group of diseases termed synucleinopathies, which includes better-studied conditions such as Parkinson’s Disease (PD) and Alzheimer’s Disease (AD). Although the exact pathogenesis has yet to be elucidated, based on epidemiological evidence, the development of MSA is largely sporadic and the etiology attributed to environmental or epigenetic factors [4, 6–8].

Several epidemiologic studies have been conducted to investigate the impact of environmental factors on the likelihood of developing MSA. An important factor to consider is cigarette smoking, given its pervasiveness in society, established impact on a macrocellular and microcellular level, and recent interest in the use of nicotine to treat neurodegenerative diseases.

The protective effect of smoking on PD is well-documented [9–12], and can be explained by the neuroprotective effect of nicotine. Although recent studies on using nicotine as a disease-modifying treatment have not yielded significant results [13], it remains a promising intervention. The impact on AD remains somewhat controversial, with different studies showing either a positive or negative relationship [14–17].

The relationship between MSA and smoking is still being debated as study findings have not produced consistent results. To address this gap in knowledge, we conducted a systematic review and a meta-analysis, which pooled data from individual studies to enhance statistical power.

## RESULTS

**Figure 1:**
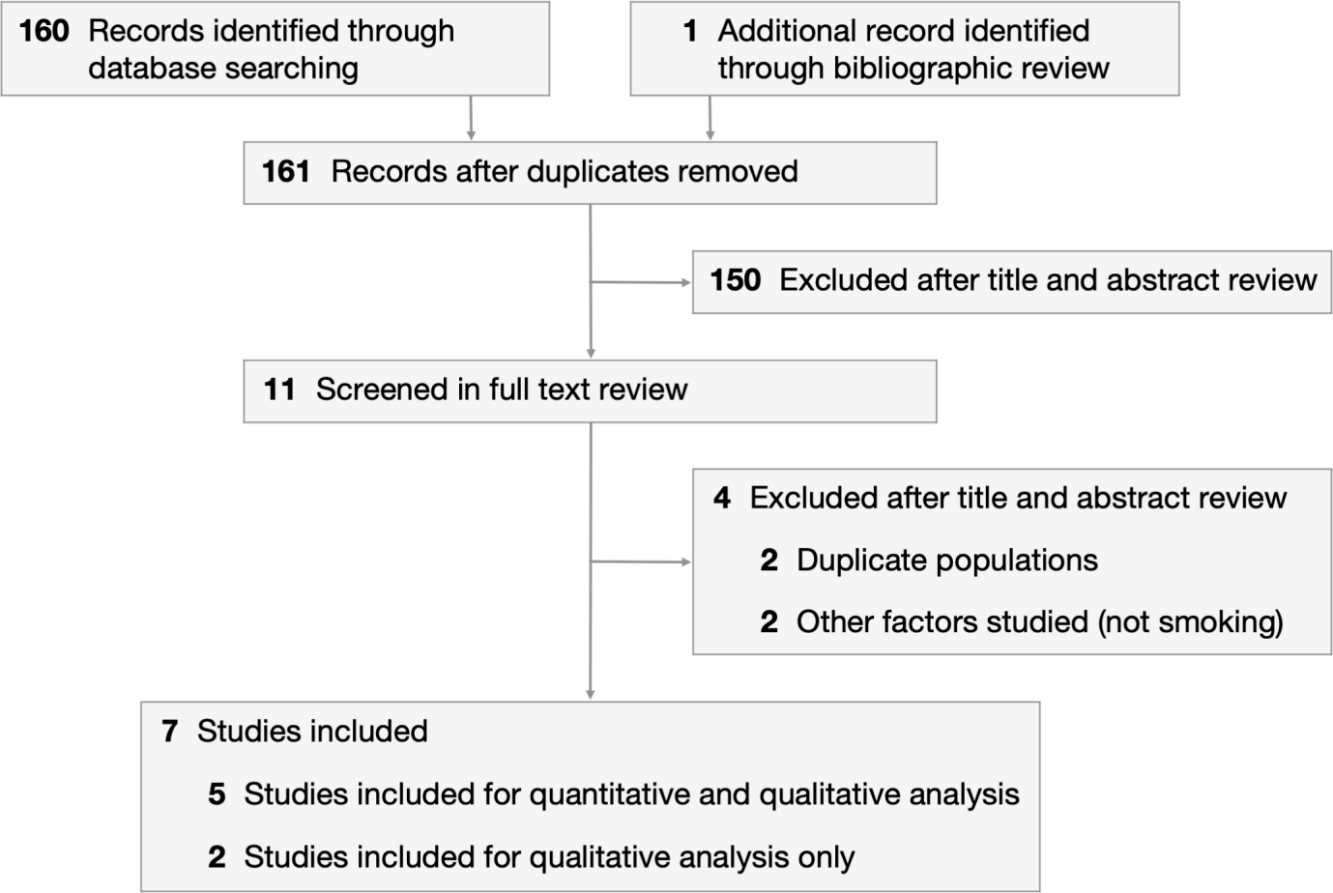
**PRISMA flowchart depicting process of study selection.**

We identified 160 articles through an electronic search on PubMed and the Cochrane Library, and 1 additional article through bibliographic review (Figure 1). After excluding 150 articles during the title and abstract review, 11 articles were screened in the full paper review. Articles were excluded if they contained duplicate populations or studied factors other than smoking. Five case-control studies were included in the meta-analysis [18–22]. This comprised 352 cases (diagnosed with MSA) and 435 healthy controls recruited from January 1994 to November 2013. Selected study characteristics are presented in Table 1. Patients were classified into never smokers and ever smokers, with 3 studies further subclassifying ever smokers into former smokers and current smokers.

**Table 1:** Study characteristics.

**Study**	**Country**	**Case: MSA Patient Population**								**Control: Healthy Control Population**				
		**Sample size, n**	**Age**	**Male Gender**	**MSA Duration**	**Never Smoker**	**Former Smoker**	**Current Smoker**	**Sample size, n**	**Age**	**Male Gender**	**Never Smoker**	**Former Smoker**	**Current Smoker**
Chrysostome 2004	France	50	67.60 ± 10.00	27	5.5 ± 2.5	31	19		50	67.40 ± 10.24	28	19	31	
Seo 2010 ^1^	South Korea	100	59.60 ± 7.90	48	3.2 ± 3.1	58	27	15	104	58.20 ± 10.10	43	69	22	13
Vanacore 2000	Italy	76	63.90 ± 7.80	41	5.0 ± 4.0	42	34		134	65.30 ± 8.50	72	58	76	
Vidal 2008 ^1^	France	71	64.76 ± 11.84	37	4.4 ± 2.6	34	11	26	71	62.99 ± 12.14	37	35	11	25
Zhou 2016 ^1^	China	55	59.33 ± 10.47	37	2.6 ± 2.2	50	1	4	76	60.41 ± 11.50	42	54	5	17
Total ^2^		352	63.0 (60.0-65.9)	54.0% (48.0%-60.0%)	4.0 (1.6-6.4)	215	137		435	62.8 (59.5-66.2)	51.0% (46.0%-57.0%)		200	
							39	45					38	55

^1^Only 3 studies subclassified ever smokers into current smokers and former smokers

^2^Continuous variables (age, MSA duration) are presented as pooled mean and 95% confidence intervals. Discrete variables (gender) are presented as percentage and 95% confidence intervals.

**Figure 2:**
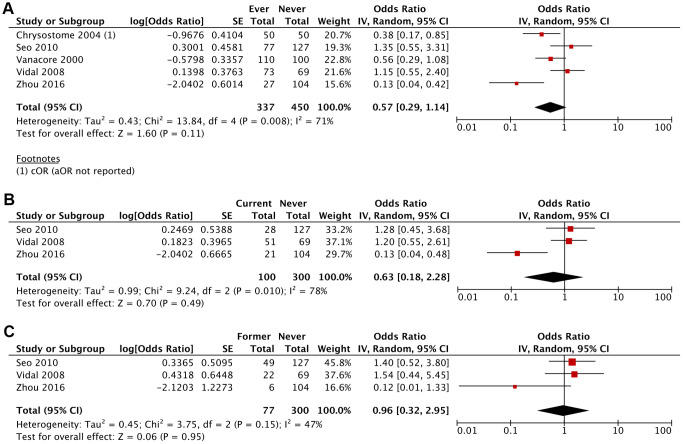
Adjusted Odds Ratio (aOR) of having MSA for: (**A**) ever smokers vs never smokers, (**B**) current smokers vs never smokers, (**C**) former smokers vs never smokers.

**Figure 3:**
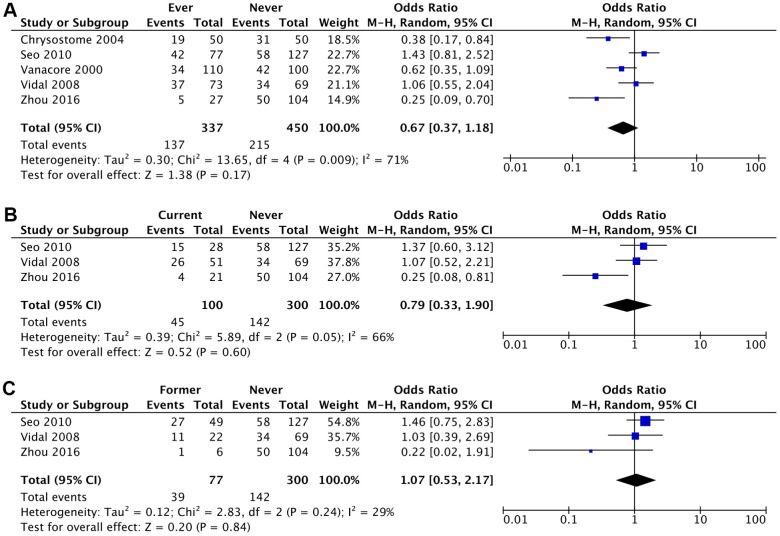
****Crude Odds Ratio (cOR) of having MSA for: (**A**) ever smokers vs never smokers, (**B**) current smokers vs never smokers, (**C**) former smokers vs never smokers.

The likelihood of developing MSA was lower amongst ever smokers (aOR 0.57; 95% CI, 0.29-1.14; *P*=0.11), current smokers (aOR 0.63; 95% CI, 0.18-2.28; *P*=0.49) and former smokers (aOR 0.96; 95% CI, 0.32-2.95; *P*=0.95) compared to never smokers, although these results were not statistically significant (Figures 2, 3 and Table 2). This association was stronger for current smokers and weaker for former smokers.

**Table 2:** Crude risk and Adjusted risk reported as crude and adjusted odds ratio.

	**Crude odds ratio (cOR) (95% CI)**	**Adjusted odds ratio (aOR) (95% CI)**
Ever Smoker vs Never Smoker	0.67 (0.37-1.18) (*P*=.17)	0.57 (0.29-1.14) (*P*=.11)
	I^2^ = 71%; τ^2^ = 0.30; χ^2^ = 13.65	I^2^ = 71%; τ^2^ = 0.43; χ^2^ = 13.84
Current Smoker vs Never Smoker	0.79 (0.33-1.90) (*P*=.60)	0.63 (0.18-2.28) (*P*=0.49)
	I^2^ = 66%; τ^2^ = 0.39; χ^2^ = 5.89	I^2^ = 78%; τ^2^ = 0.99; χ^2^ = 9.24
Former Smoker vs Never Smoker	1.07 (0.53-2.17) (*P*=.84)	0.96 (0.32-2.95) (*P*=.95)
	I^2^ = 29%; τ^2^ = 0.12; χ^2^ = 2.83	I^2^ = 47%; τ^2^ = 0.45; χ^2^ = 3.75

## DISCUSSION

There were divergent conclusions from the 5 case-control studies. Zhou et al. [18] identified current smoking as a protective factor to a statistically significant extent whereas other studies had mixed results that concluded smoking as a risk or protective factor, albeit to statistically insignificant extents. The mixed findings regarding the impact of smoking on MSA could possibly be attributed to various confounding variables including genetic differences, unknown lifestyle and environmental factors, gene-environmental interactions and other stochastic factors. We highlight the possible mechanisms linking smoking to MSA.

### Pathogenesis of smoking as a protective factor for MSA

There are several possible mechanisms in which smoking could be protective for synucleinopathies such as MSA. Given that nicotine is the major active ingredient in cigarette smoke, most of its protective effects is likely derived from the action of nicotine.

First, nicotine prevents alpha-synuclein accumulation by inhibiting the formation of alpha-synuclein fibrils and destabilizing preformed alpha-synuclein fibrils [23]. This anti-fibrillogenic and fibril-destabilizing activity was demonstrated in human-induced pluripotent stem cell-derived neurons and is postulated to arise from (i) activation of the dopamine D3 receptor and β2 subunit of acetylcholine nicotinic receptor heteromer (D3R-nAChR) and PI3K-dependent signaling pathway in dopaminergic neurons, and (ii) maintenance of protein degradation systems, such as the ubiquitin-proteasome system, that prevent accumulation of misfolded proteins [24].

Second, nicotine promotes dopaminergic neuron survival by exerting neurotrophic effects, leading to neurons with enlarged cell bodies and increased dendritic arborization [25]. It also has an inhibitory effect on apoptosis, which is a core feature in neurodegenerative diseases [26].

Third, nicotine has antioxidant effects on the central nervous system [27]. Nicotine binds to iron (Fe2+) and reduces transferrin-mediated iron uptake, which systemically reduces oxidative stress. It also activates nicotinic receptors, which have been shown to attenuate intracellular oxidative stress [28].

Fourth, nicotine is protective from certain neurotoxins, such as parkinsonism-causing ones like MPTP (1-methyl-4-phenyl-1,2,3,4-tetrahydropyridine) and methamphetamine [29, 30]. These neurotoxins have not been directly implicated in the pathogenesis of MSA, but there is likely to be an association given that the parkinsonism observed in MSA is due to neuronal loss in the substantia nigra, locus ceruleus, caudate nucleus and putamen.

Fifth, the lithium content in cigarettes may contribute to reduced risk of synucleinopathies by enhancing autophagy of damaged neurons, decreasing the aggregation and phosphorylation of alpha-synuclein, and enhancing β-catenin-mediated activity leading to increased Nurr1 expression through its ability to inhibit glycogen synthase kinase-3 β (GSK-3β) [31].

### Pathogenesis of smoking as a risk factor for MSA

Although nicotine has antioxidant effects at a biochemical level, cigarette smoking has been shown to increase general oxidative stress due to other chemicals that generate reactive oxygen species and reactive nitrogen species [32]. Oxidative stress has been shown to be a major risk factor for MSA regardless of its source [33–39], and is regarded as the final common pathway for other risk factors including pesticides, solvents and fertilizers. Smokers also have a lower intake of antioxidants compared to non-smokers [40, 41].

There have been *in vitro* studies showed that nicotine reduced levels of glutathione and elevated levels of malondialdehyde [42]. However, these studies have been criticized for simulating nicotine levels that are too high to be realistic *in vivo*.

Given that the pathogenesis of smoking as a risk factor for MSA is primarily due to other oxidative stress-generating substances in cigarettes, it is important to distinguish the impact of smoking and nicotine only on MSA.

### Alternative explanations

There have been alternative explanations proposed to address the seemingly negative association between cigarette smoking and neurodegenerative diseases. One possible reason for this epidemiological observation is the Rigg’s hypothesis, which attributes the negative association to the influence of differential survival as a result of cigarette smoking [43, 44]. Essentially, smokers with diagnosed or probable MSA are likely to have higher mortality rates compared to non-smokers, thus reflecting a proportionately greater number of non-smokers with diagnosed or probable MSA than non-smokers at any given point of time. This could lead to smoking being mistakenly interpreted as a protective factor for MSA.

It is also possible that smoking indirectly impacts the risk of developing MSA via an unknown third factor. This factor may, for example, increase the risk of MSA whilst also causing an aversion to smoking behavior [45, 46].

Data on the impact of other environmental risk factors on the development of MSA is inconclusive [47, 48]. Although some studies have shown an association between exposure to organic solvents, plastic monomers and additives, pesticides, metals and agricultural activities with increased risk of MSA [49–52], other studies have not been able to replicate the data [19, 22, 53].

### Smoking on the natural history of MSA

In a large prospective analysis, Jackson et al. found that the age of onset of MSA was 1.67 years earlier in a patient with a smoking history compared to a never smoker in a multivariate model (*P*=0.047) [54]. There was no difference in survival or disease duration (*P*=0.28), thus it is likely that smoking shifts the age of disease onset forward rather than having an impact on clinical progression. Although the results of this study ran counter to its initial hypothesis that smokers will have a later onset of disease (which is observed in PD [55]), this study does not contradict evidence that smoking is a protective factor for MSA. One possible explanation for the perceived earlier onset of MSA in smokers could be that smoking causes a transient exacerbation of the cerebellar component of the symptomatology, namely speech and gait [56]. In fact, this observation was first reported in the sentinel paper on MSA published by Graham and Oppenheimer [57]. This nicotine sensitivity is poorly understood, but may be due to significantly reduced subcortical cholinergic activity in MSA patients compared to healthy controls and PD patients, as shown in positron emission tomography studies [58–60]. The transient increase in the severity of symptoms during smoking could lead to earlier presentation and supposed earlier onset of the disease.

Smoking also did not seem to worsen the long-term clinical severity of MSA, as measured by the Unified Multiple System Atrophy Rating Scale (UMSARS) [18].

### Quantification of tobacco exposure

The degree of tobacco exposure is often quantified in terms of pack-years of cigarette smoking. Out of the five studies included in the quantitative analysis, only two studies investigated the effect of pack-years of smoking on the risk of developing MSA.

Vanacore et al. [20] dichotomized the ever smokers group into moderate smokers (≤30 pack-years) and heavy smokers (>30 pack-years) and found that the proportion of MSA patients was lower than healthy controls to a greater degree in heavy smokers (aOR 0.47; 95% CI, 0.21-1.05) compared to moderate smokers (aOR 0.64; 95% CI, 0.31-1.32). Although both results were non-statistically significant, there was the presence of a linear trend (test of departure from linearity, *P*=0.83). A decade later, Seo et al. [19] stratified tobacco exposure into 1-19 pack-years, 20-39 pack-years and ≥40 pack-years. The data was largely statistically insignificant for 1-19 pack-years (aOR 1.98; 95% CI, 0.80-4.93; *P*=0.24) and 20-39 pack-years (aOR 1.15; 95% CI, 0.52-2.58; *P*=0.78). However, data for the heavy smokers group (≥40 pack-years) seemed to contradict Vanacore et al., with a statistically significant increase in the likelihood of MSA amongst heavy smokers (aOR 3.44; 95% CI, 1.05-11.23; *P*=0.037).

### Potential limitations

There are several potential limitations observed in the case-control studies that limit the accuracy of this systematic review and meta-analysis. First, there is a possibility of clinical misclassification in the diagnosis of synucleinopathies such as MSA, PD and progressive supranuclear palsy [61, 62]. This was demonstrated in autopsies of patients who had diagnosed MSA (PPV 30%) and probable MSA (68%) [63]. The case group may thus have included patients who were misdiagnosed with MSA, thus leading to confounding results. Second, patients may untruthfully or inconsistently report their smoking status due to various reasons, such as out of fear that their standard of care will be affected [64]. This is compounded by the possibility of recall bias where cases are better able to recall their smoking status due to greater investment in the studies.

Furthermore, the results may not be generalizable across all populations and geographic regions, given that the all studies were conducted in either Asia or Europe.

### Future directions

To address the limited sample population due to the rarity of MSA, large scale multicenter collaborative prospective studies across various populations are needed to determine the presence of a cause-effect relationship, particularly the association between heavy smokers or high nicotine dosages, and MSA. This will strengthen the credibility of evidence by reducing the probability of recall bias from case-control studies. Functional studies to examine the effect of smoking (nicotine and other constituents) in human cell lines and animal models of MSA can provide new insights into the underlying path-mechanism. Lastly, the impact of exposure to nicotine only on MSA should be further investigated, such as in individuals who are prescribed nicotine replacement therapy in the form of gum, transdermal patches or nasal spray. If shown to be protective, there is potential for nicotine to slow the progression of MSA and be a disease-modifying treatment.

## MATERIALS AND METHODS

This study was conducted in accordance with the Preferred Reporting Items for Systematic Reviews and Meta-Analyses (PRISMA) and Meta-Analysis of Observational Studies in Epidemiology (MOOSE) guidelines [65, 66].

### Search strategy and selection criteria

We searched PubMed and the Cochrane Library from inception to July 7, 2019. The following terms were included in the search strategy to encompass all variants of MSA: “Multiple system atrophy”, “Shy-Drager syndrome”, “Olivopontocerebellar atrophies” and “Striatonigral Degeneration”. The search was conducted using the above terms and “Smoking”, “Nicotine”, “Tobacco” and “Cigar*”. Gray and bibliographic searching was done by reviewing the references of included studies and related review articles. To limit publication bias, we considered meeting abstracts and unpublished online data if there were sufficient results to analyze.

Inclusion criteria were (i) case-control studies, (ii) MSA cases diagnosed with clinical criteria, (iii) reported data for smoking as an environmental risk or protective factor, and (iv) sufficient raw data available for analysis. Excluded from the meta-analysis were nonhuman studies and case reports. The eligibility of studies was independently assessed by two review authors (FST, XD) and disagreements were resolved by consensus or appeal to a third author (EKT). The full electronic search strategies are presented in the Supplementary Table 1.

### Data extraction

Two review authors (FST, XD) independently extracted data from included studies. We extracted study characteristics such as the country, data source, period, inclusion and exclusion criteria, study aims, method of MSA diagnosis, patient demographics and the number of MSA patients and healthy controls. Outcome data comprise the number of smokers and non-smokers amongst MSA patients and healthy controls, duration of MSA, severity of MSA and quantification of tobacco exposure. We extracted absolute, relative, crude and adjusted effects.

### Risk-of-bias and quality assessment

The risk-of-bias and study quality was assessed using the Joanna-Briggs Institute (JBI) Critical Appraisal Tool for Case-Control Studies as well as the Newcastle-Ottawa Scale. The detailed analysis of each study is presented in Supplementary Tables 2, 3.

### Statistical analyses

We performed frequentist, pairwise meta-analysis in RevMan version 5.3 (The Cochrane Collaboration). All meta-analytical techniques were done assuming the random-effects model, which accounts for variance across included studies. We presented between-study statistical heterogeneity using I^2^, τ^2^, and χ^2^ statistics. Pooled relative effects were obtained and presented as odds ratios (OR) using the Mantel-Haenszel method. Although interpretation of disease risk was mainly driven by 95% confidence intervals, we also considered two-sided *P*<.05 to indicate nominal statistical significance.

## CONCLUSIONS

While there is a suggestion that cigarette smoking may be a protective factor for MSA, the scientific evidence of this relationship is still weak. Further clinical and laboratory studies could unravel the functional evidence of such a cause and effect links. Nicotine studies as a potential disease-modifying treatment for MSA should be further pursued.

## Supplementary Material

Supplementary Tables
