# Supplementary material for: Multiple System Atrophy (MSA) and smoking: a meta-analysis and mechanistic insights
Source: Aging (Albany NY). 2020 Nov 7;12(21):21959–70. doi: 10.18632/aging.104021 (PMC7695394; doi:10.18632/aging.104021)
Supplement: Supplementary Tables [file aging-12-104021-s001..pdf]

SUPPLEMENTARY TABLES

Supplementary Table 1. Search Strategy.

#1	Multiple System Atrophy[All Fields] OR MSA[All Fields] OR Shy-Drager Syndrome[All Fields] OR Shy Drager Syndrome[All Fields] OR olivopontocerebellar atrophy[All Fields] OR OPCA[All Fields] OR striatonigral degeneration[All Fields]
#2	Smoking[All Fields] OR Nicotine[All Fields] OR Cigar*[All Fields] OR Tobacco[All Fields]
#3	#1 and #2

Supplementary Table 2. The Joanna Briggs Institute Critical Appraisal Tool for Case-Control Studies.

Code	Were the groups comparable other than the presence of disease in cases or the absence of disease in controls?	Were cases and controls matched appropriately?	Were the same criteria used for identification of cases and controls?	Was exposure measured in a standard, valid and reliable way?	Was exposure measured in the same way for cases and controls?	Were confounding factors identified?	Were strategies to deal with confounding factors stated?	Were outcomes assessed in a standard, valid and reliable way for cases and controls?	Was the exposure period of interest long enough to be meaningful?	Was appropriate statistical analysis used?
Zhou 2016	Yes	Yes	Yes	No	Yes	Yes	Yes	Yes	Unclear	Yes
Seo 2010	Yes	Yes	Yes	No	Yes	Yes	Yes	Yes	Yes	Yes
Vanacore 2000	Yes	Yes	Yes	No	Yes	Yes	Yes	Yes	Yes	Yes
Chrysostome 2004	Yes	Yes	Yes	No	Yes	No	No	Yes	Unclear	Yes
Vidal 2008	Yes	Yes	Yes	No	Yes	Yes	Yes	Yes	Unclear	Yes

Supplementary Table 3. The Newcastle-Ottawa Quality Assessment Scale for Case-Control Studies.

Code	Selection				Comparability	Exposure			Total
	Definition of Cases	Representativeness of Cases	Selection of Controls	Definition of Controls		Ascertainment of Exposure	Method of Ascertainment	Non-Response	
Zhou 2016	1	1	1	1	2	0	1	0	7
Seo 2010	1	1	1	1	2	0	1	0	7
Vanacore 2000	1	1	1	1	2	0	1	0	7
Chrysostome 2004	1	1	1	1	1	0	1	0	6
Vidal 2008	1	1	1	1	2	0	1	0	7
